# Cellulose Nanocrystals as Nucleating Agents in Lignin-Based Rigid Polyurethane Foams

**DOI:** 10.1021/acsomega.6c07686

**Published:** 2026-09-25

**Authors:** Kelvin Techera Barbosa, Raphael Duarte Gimnecki, Sandro Campos Amico, Rafael de Avila Delucis

**Affiliations:** † PPGE3M, Federal University of Rio Grande do Sul, Porto Alegre 90040-060, Brazil; ‡ PPGCEM, Federal University of Pelotas, Pelotas 96010-610, Brazil; § PPGEP, Federal University of Goiás, Aparecida de Goiânia 74971-451, Brazil

## Abstract

This study investigates the incorporation of cellulose nanocrystals (CNC) into rigid polyurethane foams (RPUF) derived from lignin polyol. CNC promoted cellular refinement consistent with a heterogeneous nucleation mechanism, reducing average cell size by up to 18% and improving cellular uniformity, particularly at 0.2% and 0.4% concentrations. Fourier-transform infrared spectroscopy (FTIR) confirmed hydrogen bonding between CNC hydroxyl groups and the polyurethane matrix. Thermogravimetric analysis revealed a shift in the temperature at 10% mass loss from 272 °C (control) to 282–285 °C (0.2–0.4% wt % CNC), while DSC showed an increase in glass transition temperature (*T*_g_) obtained by DSC from 31.5 °C for the neat RPUF to 58.2 °C for RPUF_0.2%. SEM micrographs confirmed more homogeneous cell distribution at low CNC loadings, whereas higher concentrations (0.6–0.8%) led to agglomeration and irregular cells. Apparent density increased by ∼7% at 0.4 wt % CNC, correlating with enhanced compressive strength (+12%) and storage modulus (+15%). The improvement in compressive performance was present after normalization by apparent density, indicating that the mechanical response was not exclusively density driven. Overall, CNC addition improved the structure and thermo-mechanical performance of lignin-based RPUF, being a sustainable route to high-performance foams for energy-efficient applications.

## Graphical abstract

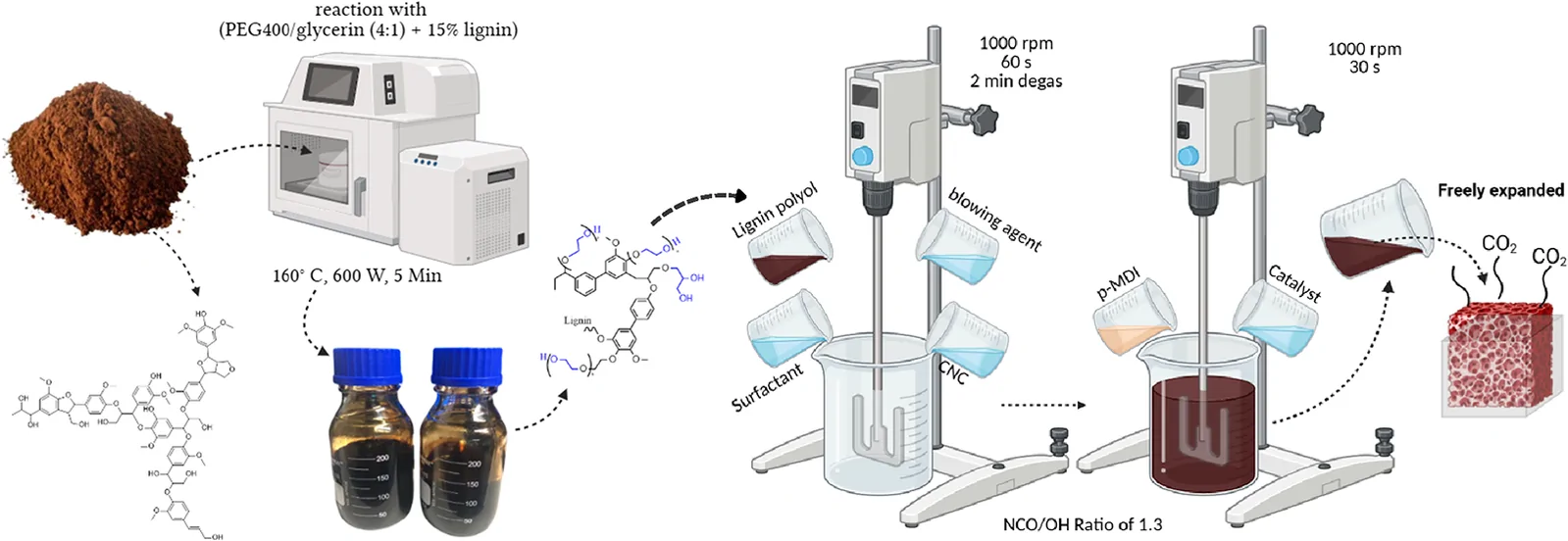

## 1 Introduction

The global polyurethane foam market size was valued at USD 49.5 billion in 2023 and is projected to reach USD 67.8 billion by 2028, growing at a 6.5% CAGR from 2023 to 2028. The major reasons for the growth of the polyurethane foams market are the demand for energy-efficient solutions. Rigid polyurethane foam (RPUF) is an excellent thermal insulator, used in construction for insulation purposes. With increasing concerns about energy conservation and sustainability, builders and homeowners are turning to polyurethane foam to enhance the energy efficiency of buildings, reducing heating and cooling costs.^1^

To further lessen the environmental impact of RPUF, biobased aromatic or aliphatic polyols with different molecular structures have been developed to replace their fossil-based equivalents. These biobased polyols are increasingly used in industry to produce sustainable materials. Gondaliya and Najad (2021)^2^ synthesized RPUF partially substituting (20% by weight) petroleum-based polyols with 15 different types of lignin-based polyols. High levels of mechanical properties, including compression force deflection, compression modulus, tensile strength and tear resistance, were also reached for lignin-based RPUF.^3^ RPUF produced with lignin polyols may also have large and anisotropic cells, which may require additives such as catalysts and chain extenders to reach a more uniform, denser and finer cell foam structure.^4^

Besides of the cited additives, the incorporation of fillers into RPUF has become a widely adopted strategy to tailor their properties, reduce costs, and expand their range of applications.^5^ Fillers can influence the final characteristics of the RPUF at multiple levels, including its density, mechanical strength, thermal behavior, flammability, and aesthetical features.^6–8^ Depending on their nature and interaction with the polymer matrix, fillers may act through different physical–chemical mechanisms.

One of the most common roles of fillers is to enhance the mechanical performance of RPUF. Fibers such as glass, carbon, or natural fibers reinforce the foam structure by transferring stress from the soft polymeric matrix to the stiff reinforcement.^9^ In RPUF, these fillers improve compressive strength, stiffness, and dimensional stability by supporting the cell walls and struts.^10^

On the other hand, certain fillers function as flame retardants or protective barriers. Layered silicates, expandable graphite, and metal hydroxides form physical shields that reduce the diffusion of heat and gases during combustion.^11^ Some fillers also release water or inert gases, suppressing flammable volatiles and slowing down fire propagation.^12^ Lightweight fillers, such as hollow glass or ceramic microspheres, are used to lower the density of RPUF.^13,14^ These preformed hollow particles decrease overall foam weight while maintaining compressive performance, which is particularly beneficial in automotive and aerospace components.^15^

Titanium dioxide (TiO_2_), zinc oxide (ZnO), and similar oxides are used to enhance UV stability and optical performance.^16^ By scattering or absorbing radiation, they mitigate photo-oxidative degradation and preserve the aesthetic and structural integrity of RPUF. Fillers like fumed silica, organo-modified clays, or micro fibrillated cellulose adjust viscosity and viscoelastic properties of the foam precursor mixture^17^ Increased viscosity stabilizes bubble growth, reduces cell collapse, and improves processing consistency.

Finally, a specific class of fillers serves as nucleating agents in RPUF. Nanoparticles with high surface energy and good dispersion, such as cellulose nanocrystals (CNCs), silica nanoparticles, or nano clays, provide heterogeneous nucleation sites for bubble formation.^18^ These fillers reduce the energy barrier for nucleation, refine cell size, increase cell density, and generate a more uniform morphology.^18^ The resulting microstructure enhances thermal insulation, mechanical strength, and dimensional stability of RPUF.^19^

Finally, a specific class of fillers serves as nucleating agents in RPUF. Cell nucleation in foams occurs when the gas generated by the reaction of isocyanate with water or by chemical/physical blowing agents overcomes the energetic barrier to form a new dispersed phase (bubble).^20^ Several recent publications have reported that fillers with abundant hydroxyl groups on their surface can interact through hydrogen bonding with polyols or urethane segments, thereby anchoring the particles within the polymer matrix.^21^ This partial compatibility facilitates the dispersion of cellulose nanocrystals (CNCs) and enables them to act as “anchors” that stabilize the growing gas nuclei.^22^ Moreover, these dispersed fillers behave as a network of physical barriers within the viscous matrix, hindering bubble coalescence and collapse during foam growth.^17^

In this context, cellulose nanocrystals (CNCs) have emerged as one of the most promising biobased nanomaterials due to their abundance, renewable origin, high crystallinity, and remarkable surface functionality.^22,23^ Typically extracted from lignocellulosic biomass through acid hydrolysis, CNCs present a rod-like morphology with dimensions in the nanometre range and a high aspect ratio.^23^ Their surfaces are rich in hydroxyl groups, which provide opportunities for chemical modification as well as strong hydrogen bonding interactions with polymer matrices.^24^ These intrinsic features make CNCs highly attractive as reinforcing agents in a wide range of polymeric systems.^22^

In the context of RPUF, CNCs have been studied mainly as nanofillers to enhance mechanical properties, thermal stability, and dimensional integrity.^25^ Previous research has demonstrated that their addition can lead to finer cell structures, reduced anisotropy, and improved compressive performance.^25^ However, the underlying mechanism behind these effects is not yet fully understood. It has been suggested that CNCs may act as heterogeneous nucleating agents during foam expansion, owing to their high surface energy and ability to interact with polyols or urethane linkages through hydrogen bonding.^24^ This hypothesis is supported by observations of increased cell density and smaller average cell size when CNCs are incorporated.^17,25^

Previous studies have demonstrated that cellulose nanocrystals and other forms of nanocellulose can modify the cellular structure of RPUF by providing heterogeneous nucleation sites, reducing cell size, increasing cell density, and improving cellular uniformity. Septevani et al.^26^ reported that CNC incorporation promoted cell refinement and improved thermal insulation of RPUF. Subsequent studies have shown that the magnitude of this nucleating effect depends on nanocellulose concentration, particle dispersion, surface chemistry, polyol composition, and processing conditions.

The dispersion of nanocellulose in polyol is particularly important because well-dispersed particles can provide a larger number of effective nucleation sites, whereas aggregation may produce heterogeneous cellular structures. Haridevan et al.^27^ demonstrated that nanocellulose loading and dispersion quality significantly affect the rheological behavior of polyol dispersions, affecting processing and the resulting foam morphology. Some authors^28^ highlighted that the high specific surface area and hydroxyl-rich surface of CNC favor hydrogen-bonding interactions, while increasing the tendency of the particles to aggregate. Similarly, Samyn and Cosemans^29^ showed that the behavior of nanocellulose in polymeric matrices depends on particle morphology, concentration, compatibility, dispersion quality, and the formation of interconnected structures near the percolation threshold.

Interactions between CNC and polyurethane may also contribute to the restriction of polymer-chain mobility, physical cross-linking, and stress transfer.^30^ attributed the improved performance of PU–CNC composites to hydrogen bonding between the hydroxyl groups on the CNC surface and carbonyl-containing groups of polyurethane, leading to the formation of a dynamic physical network. The importance of hydrogen-bond-mediated network formation in cellulose-containing polymer systems has also been demonstrated by.^31–33^ Although these studies did not specifically investigate nucleation in RPUF, they support that molecular interactions may accompany CNC incorporation and influence polymer-chain mobility, interfacial behavior, and material performance.

As a result, while CNCs are widely recognized as multifunctional nanofillers with strong potential to influence the cellular architecture of RPUF, further systematic investigations are required to confirm and explain their nucleating mechanism.^26^ In this study, the effect of CNC addition on cellular morphology, thermal behavior, rheological properties, and mechanical performance of rigid polyurethane foams was investigated, with particular attention to its potential role in heterogeneous nucleation.

## 2 Experimental Section

### 2.1 Lignin-Based Polyols

Kraft lignin powder was kindly donated by Suzano Papel e Celulose Company (Brazil), which obtained this product by a process like the Lignoboost method. To produce the polyol, a ratio of 80/20 (w/w) (PEG400/glycerine) and 10 (%/w) of additional lignin were used in a MARS 6 synthesis preparation microwave (CEM Corporation) with an Easy Prep reactor, at a temperature of 160 °C, with 600 W for 5 min. The reaction was carried out under constant stirring. Once the reaction time had elapsed, the reactor cooling system was activated to cool the vessel to 50 °C. The chemicals were selected and prepared as described in a previous study.^4^

Steady-shear rheological measurements were performed using an Anton Paar MCR 101 rheometer at 25 °C, equipped with a 25 mm parallel-plate geometry and a gap of 1 mm. The shear rate varied from 0.01 to 1000 s^–1^ and subsequently decreased back to 0.01 s^–1^. The resulting viscosity and shear-stress curves were used to evaluate the steady-shear flow behavior of the lignin-based polyols containing different CNC concentrations. Dynamic viscosity was measured using a Brookfield SP-920 viscometer using a 50 mL sample at 25 ± 1 °C with an L3 spindle at 60 rpm. The reported viscosity corresponds to the mean of three measurements. In addition, each polyol was placed in a volumetric pipet to measure its volume and then weighed to determine its density and used to calculate the kinematic viscosity.

### 2.3 Lignin-Based RPUF

The CNC used in this study was supplied by CelluForce Industry, Montreal, QC, Canada, in the form of spray-dried powder. The product has a whitish color, apparent density between 0.4 and 0.6 g/cm^3^ and moisture content ≤ 6.0%. The particle size of the powder is within 1–50 μm, while dynamic light scattering analysis indicates an average particle size of 150 nm. Prior to incorporation into the polyol, the CNC powder was dried in a vacuum oven at 50 °C for 48 h under an absolute pressure of 50 mmHg. The lignin-based polyol (66.97 p/g) was kept at room temperature. Then, the CNC was added to reach contents of 0.2, 0.4, 0.6 and 0.8% by mass in relation to the total mass of the formulation, and dispersed in the polyol using a propeller-equipped RW20 Package digital mechanical stirrer at 1000 rpm for 120 s. Subsequently, surfactant (1.88 p/g) and catalyst (0.37 p/g) were added in that order, and the complete mixture was again mechanically homogenized at 1000 rpm for 120 s.^26^ Then, polymeric MDI (68.82 p/g) was added manually, and the formulation was mechanically stirred for another 30 s before pouring into an iron mold. The amount of polymeric MDI was adjusted to maintain an NCO/OH ratio of 1.3. After 24 h, the RPUF was demolded and left to cure at room temperature for 2 weeks.

The molded RPUF specimens were also analyzed using a Fourier transform infrared spectrophotometer (FTIR) (Thermo-Nicolet 5700, Thermo electron Corp) equipped with a diamond attenuated total reflection (ATR) accessory. Spectra were recorded on the reflectance mode in the wavenumber range of 500–4000 cm^–1^ over 64 scans with a resolution of 4 cm^–1^, Thermogravimetric (TG) curves were obtained under nitrogen atmosphere at a heating rate of 10 °C.min^–1^ using a Q50 equipment (TA Instruments) according to ASTM D7582, from room temperature to 800 °C. Differential scanning calorimetry (DSC) was performed in a Q20 calorimeter (TA Instruments) under nitrogen atmosphere at 50 mL.min^–1^ flow rate. The samples were heated, cooled and heated again in the −85 to 240 °C range, at a rate of 10 °C.min^–1^, Water uptake measurements were performed on ten specimens (dimensions: 5.0 × 5.0 × 2.5 cm^3^) per group, with the smallest dimension oriented in the foam-rise direction. The specimens were fully immersed in distilled water at room temperature (≈20 °C), in accordance with ASTM D570. Mass gain was monitored at 30 min intervals for up to 300 min. Before weighing, excess surface water was removed using filter paper.

Morphology of the RPUF was analyzed perpendicular to the rise direction by scanning electron microscopy (SEM) in MA10 equipment (Zeiss Evo brand, Oberkochen/Germany) operating at 3 kV. Average cell width, length, and anisotropy index were measured using ImageJ software (Version 1.53t 24), as described by Delucis and co-workers [17]. A Mark Olympus optical microscope (OM) (2.5× magnification) was utilized to analyze the transverse plane in relation to the foam rise. The samples were carefully cut, glued to stubs and coated with a thin layer of gold. The average cell diameter (CD) (eq 1) and the anisotropic index (AI) (eq 2) were estimated using 12 measurements per family (3 samples with 5 images each). The measurements were taken from the SEM images using ImageJ software. In addition, the mean projected cell area (Ap) was calculated for each formulation by approximating its two-dimensional profile as an ellipse, using the measured cell length and height as the principal dimensions, according to eq 3.

1 $$\mathrm{CD}=\frac{\left(c\mathrm{ell width}+c\mathrm{ell length}\right)}{2}$$

2 $$\mathrm{AI}=\frac{\left(c\mathrm{ell length}-c\mathrm{ell width}\right)}{c\mathrm{ell length}}$$

3 $$\mathrm{Ap}=\frac{\left(\pi \times c\mathrm{ell length}\times c\mathrm{ell height}\right)}{4}$$

This method followed ISO 2896 ^34^ except for the dimensions, which were 5.0 × 5.0 × 2.5 cm^3^. Apparent density of ten cubic samples (2.5 cm sides) per group was obtained using an analytical scale and a digital calliper. These same samples were tested in compression using a universal testing machine (Emic equipment, model 23–5D) with the load applied parallel to the direction of expansion. The test speed was 2.5 mm.min^–1^, and the compressive strength was read when the load remained constant (close to 3.3 mm of machine displacement), in accordance with ASTM D1621. Specific compressive strength and specific compressive modulus were calculated by dividing the respective mechanical property by the apparent density for each formulation, given as kPa·cm^3^·g^–1^ and MPa·cm^3^·g^–1^, respectively.

The storage modulus (E′), loss modulus (E″), tan δ and glass transition temperature (*T*_g_) were determined based on curves obtained by dynamic mechanical analysis (DMA) (on a TA Instruments model 2980), operating with a standard dual cantilever clamp. The tests were carried out on prismatic samples (dimensions: 3.5 × 1.2 × 4.5 mm^3^, smallest dimension in the direction of expansion) under an inert atmosphere of N2, a frequency of 1 Hz and a deformation of 10%, over a temperature range of −50 to 100 °C with a heating ramp of 10 °C.min^–1^ The effectiveness of the reinforcement (*C*) was calculated using eq 4, as per Pothan et al.^35^

4 $$C=\frac{(\frac{E^{\prime }g}{E^{\prime }r})CNCRPUF}{(\frac{E^{\prime }g}{E^{\prime }r})RPUF}$$

where E′g and E′r are the storage moduli related to the glassy and rubbery regions, respectively. Some results were subjected to ANOVA tests, and whenever the null hypothesis was rejected, Tukey test was used to compare the means.

## 3 Results and Discussion

Figure 1A shows the apparent viscosity of the lignin-based polyols as a function of shear rate. The viscosity of each formulation remained relatively constant within the investigated shear-rate range, and Figure 1B shows an approximately linear relationship between shear stress and shear rate on a log–log scale, both indicating approximately Newtonian steady-shear flow behavior of the formulations. The curves showed similar profile, indicating that CNC incorporation did not alter the general flow regime of the polyol under the evaluated conditions.^36^

**Figure 1:**
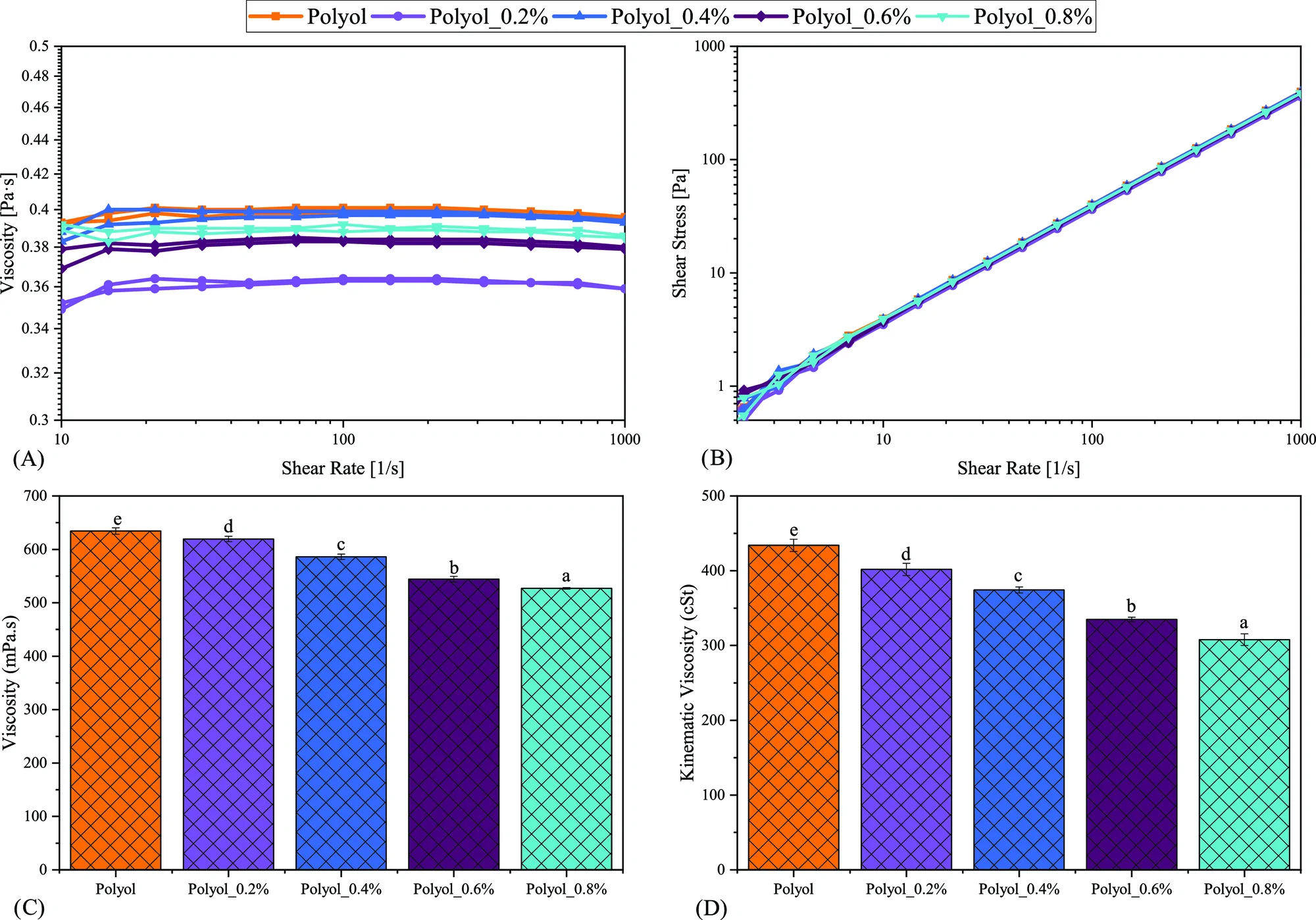
Shear rate viscosity curve (A), shear stress vs shear rate curve (B), dynamic viscosity (C) and kinematic viscosity (D) of the lignin-based polyols.

Both dynamic viscosity (Figure 1C) and kinematic viscosity (Figure 1D) decreased as the CNC concentration increased. All formulations exhibited approximately Newtonian behavior under the evaluated conditions. Haridevan et al.^27^ also reported that a polyol dispersion with low nanocellulose concentration exhibited lower viscosity than that the neat polyol, and near-Newtonian behavior, which was attributed to effective particle dispersion promoted by hydrodynamic forces. At low concentrations, the particles remain below the percolation threshold and do not form a sufficiently interconnected network to restrict flow of the polyol.^27^ The rheological effect of nanocellulose depends on its concentration, morphology, surface characteristics, dispersion quality, and interactions with the surrounding medium. CNCs have a high specific surface area and hydroxyl-rich surfaces that favor hydrogen bonding. However, these interactions only produce a marked increase in viscosity when particle–particle contacts generate a continuous or percolated structure. Therefore, the CNC concentrations used in this work were insufficient to form a strong interconnected network in the lignin-based polyol..^28,29,37,38^

Figure 2 shows FTIR spectra of the control, and of the additions of 0.2%, 0.4%, 0.6%, 0.8% by weight of CNC, all the samples showed infrared spectra typical of PU foam. For example. The presence of urethane bonds can be observed from peaks at 3647 cm^–1^ (free N–H), 3200 cm^–1^ (hydrogen-bonded N–H), 1212 cm^–1^ (C–N) and 1059 cm^–1^ (C=O).^4,39^

**Figure 2:**
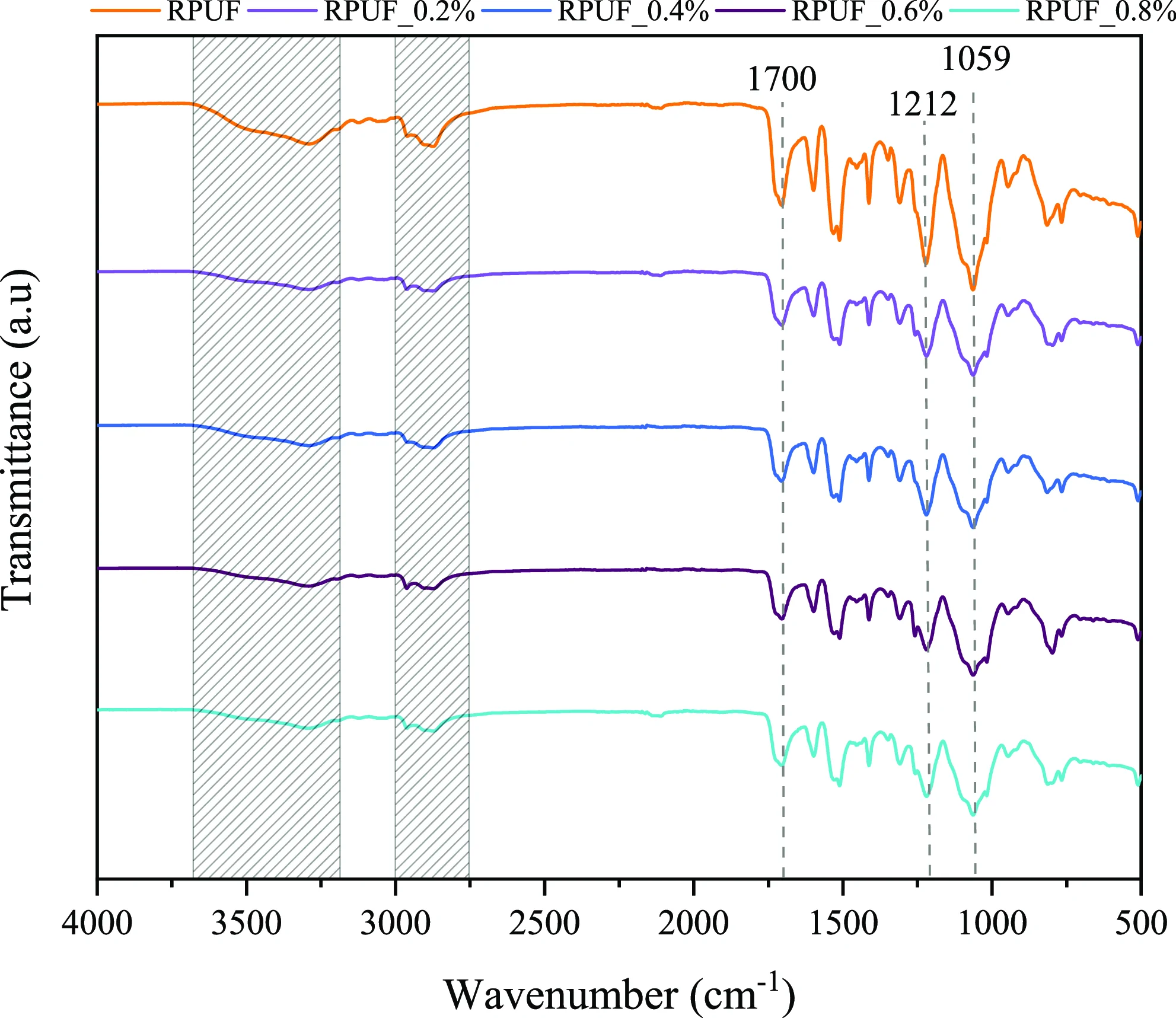
FT-IR spectra of the lignin-based RPUF.

With the addition of CNC in increasing concentrations, some notable changes were observed in the FTIR spectra. The intensity of the O–H and N–H stretching bands at 3200–3647 cm^–1^ tended to decrease slightly with increasing CNC content. This change can be interpreted as the possible formation of hydrogen bonds between the hydroxyl groups of cellulose and the N–H or C=O groups of the polyurethane matrix, consuming more N–H groups in the formation of urethane, which would result in a restriction of the mobility of these groups and, consequently, a decrease in their absorption intensity.^40^

The addition of CNC decreased the flexible aliphatic C–H groups of the polyol identified with the 3000–2755 cm^–1^ band vibration, as well as reducing the 1700 cm^–1^ peak. This may indicate that the intense interactions between the hydroxyl groups of the CNC and the carbonyls of the urethane are altering the nature of the C=O vibration.^41,42^

The consumption of alcoholic hydroxyl groups (O–H) from the CNC or polyol, which are highly reactive, can occur due to the reaction with isocyanates, reducing the amount of these free groups and consequently decreasing the intensity of these bands. This structural reorganization and the formation of a denser network affect the stretching vibrations of the C–O bonds (1059 cm^–1^), reflected in the decrease in the corresponding peaks in infrared spectroscopy. Therefore, the presence of CNC not only dilutes the lignin units, but also reorganizes their C–O ether structure (1212 cm^–1^), directly impacting the physicochemical properties of the resulting material.^43^

The intensity of these changes depends on the concentration of CNC, the type of polyol and the incorporation route, and can have an impact on mechanical, thermal and optical properties and biocompatibility.^41,42,44,45^

Figure 3 shows the TG and DTG curves for RPUF. The temperature at 10% mass loss increased from 272.6 °C for the neat RPUF to 282.3–285.3 °C for the CNC-containing foams.^42^ The 50% degradation of all modified CNCs was observed within the temperature range of 378–376 °C, which is a higher temperature than the 372 °C control foam,^41^ but the 0.6% and 0.8% foams degraded more as the temperature increased, which is due to the excess reinforcement in the foam. In addition, the neat RPUF reached 10% mass loss at a lower temperature than all CNC-containing formulations, with the highest T10% values observed for RPUF_0.2% and RPUF_0.4%.^42^ The main mass-loss temperatures are compiled in Table 1.

**Figure 3:**
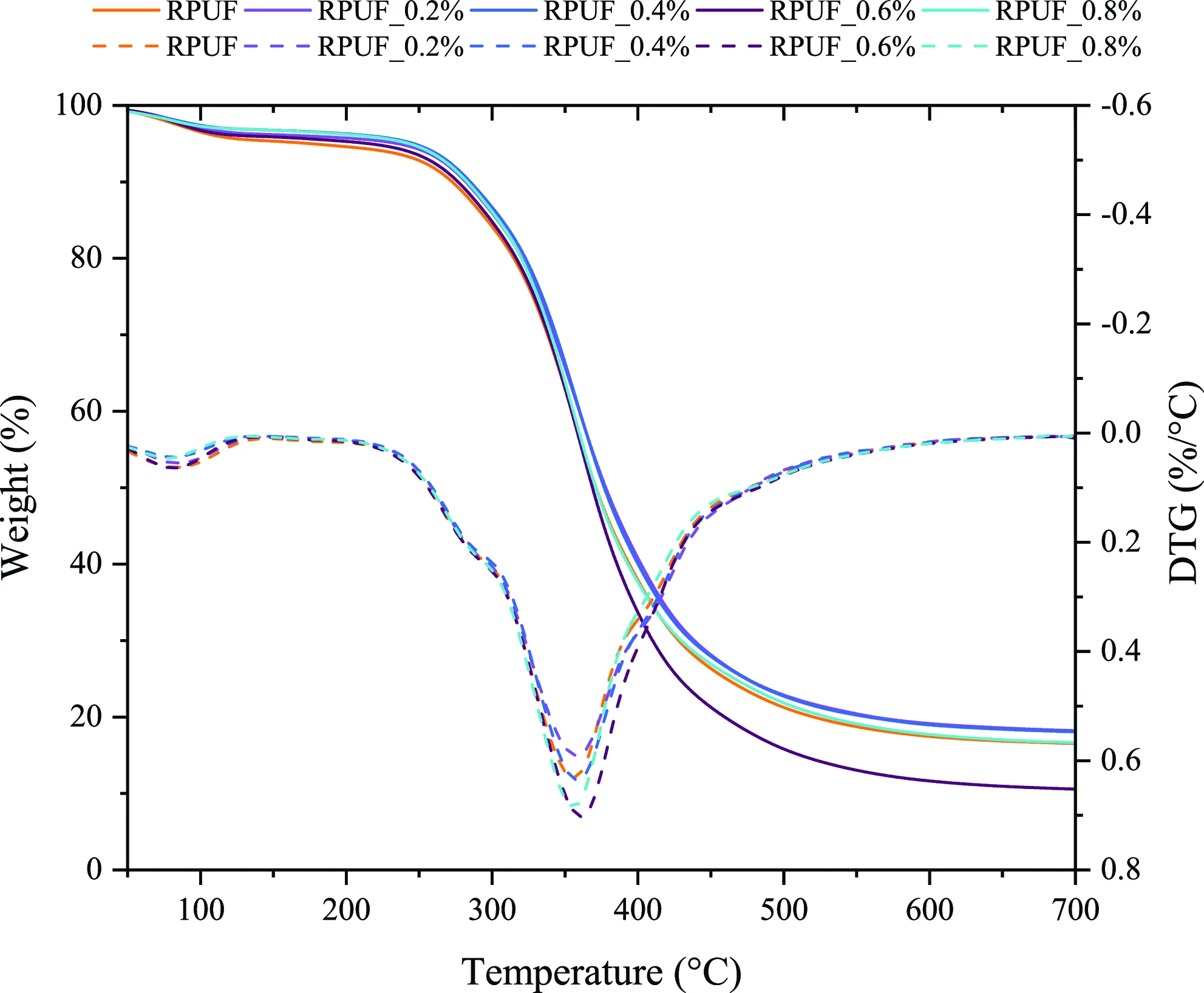
TG/DTG curves of the lignin-based RPUF.

**Table 1:** Temperatures Corresponding to Different Mass-Loss Levels for the Lignin-Based RPUF

	*T*_2%_	*T*_10%_	*T*_50%_	*T*_80%_
RPUF	61.8	272.6	372.1	515.7
RPUF_0.2%	79.7	282.8	378.5	560.9
RPUF_0.4%	84.8	285.3	377.3	550.1
RPUF_0.6%	75.9	282.3	376.0	457.6
RPUF_0.8%	78.4	282.8	376.8	516.8

The second-heating DSC curves presented in Figure 4B show glass transition events within ca. 30–60 °C. The neat RPUF exhibited a *T*_g_ of 31.5 °C, whereas the incorporation of CNC increased *T*_g_ to 58.2, 53.3, 54.9, and 54.0 °C for RPUF_0.2%, RPUF_0.4%, RPUF_0.6%, and RPUF_0.8%, respectively. The increase in *T*_g_ indicates reduced segmental mobility of the polyurethane chains, especially for the RPUF_0.2%, which may be associated with stronger interactions between CNC hydroxyl groups and the polyurethane network, increasing chain-mobility restriction. The addition of CNC weight resulted in a slight increase in *T*_g_ at all percentages. This behavior can be compared to previous studies indicating that small amounts of nanofillers can act as nucleating agents, promoting a finer and more homogeneous cellular structure. This more compact structure tends to restrict the movement of polymer chains, increasing *T*_g_.

**Figure 4:**
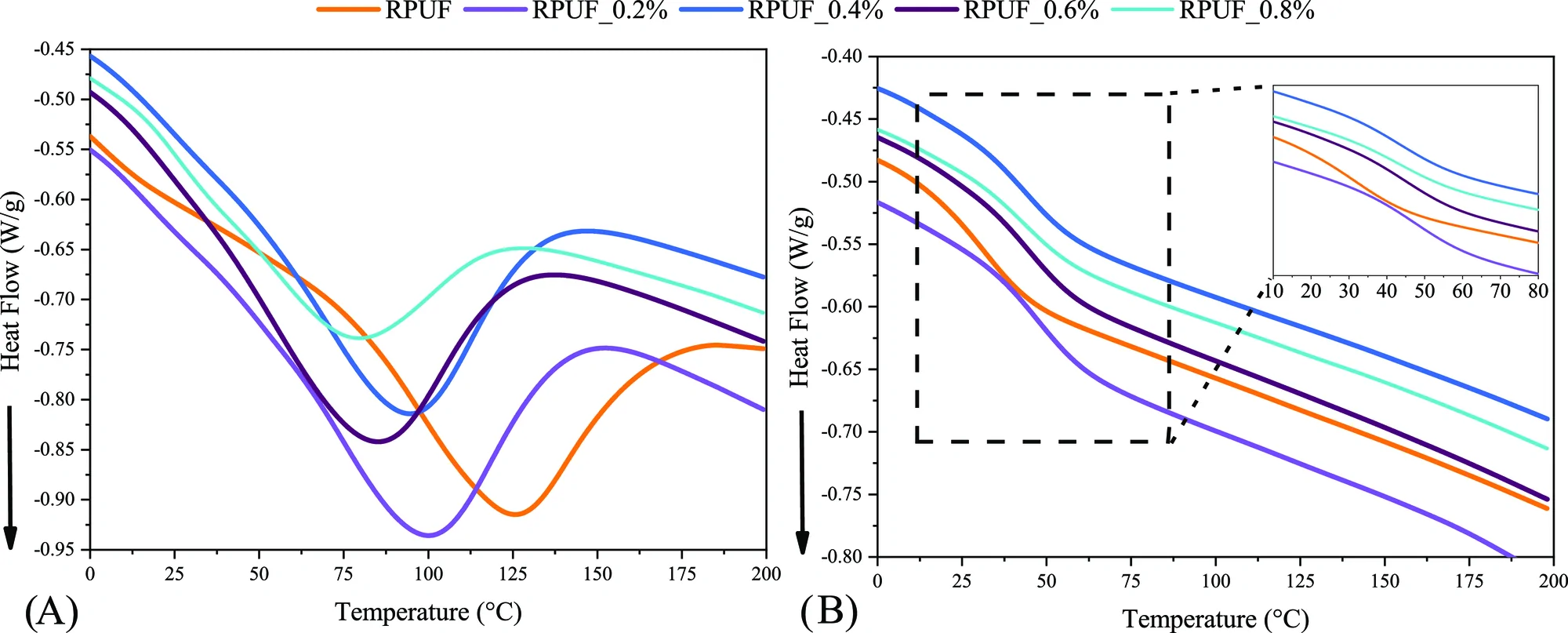
DSC curves obtained during the first (A) and second (B) heating of the lignin-based RPUF.

This finding suggests an increased dispersion of the nanocrystals in the polymer matrix, maximizing the interactions between the hydroxyl groups, abundant in CNC, and the polyurethane network. The high concentration of hydroxyl groups in CNC Favors the formation of hydrogen bonds, which can increase the cross-linking density or create an interpenetrating polymer network, contributing to the material’s greater rigidity. Previous studies have also shown that incorporating lignin into PU foams can result in greater rigidity due to increased cross-linking reactions and the formation of rigid segments.^46,47^ However, higher concentrations of CNC, such as 0.6% and 0.8% by weight, induced a slight decrease in *T*_g_ compared to the 0.2% CNC sample. This behavior is often attributed to the agglomeration of nanofillers at high concentrations, a common challenge in composites.

Particle agglomeration can create structural defects and reduce the effective surface area for interaction with the matrix, compromising the reinforcement capacity and, consequently, the uniform restriction of the mobility of the polymer chains. This observation is consistent with reports in the literature describing a reduction in the density of RPUF with increasing lignin content, associated with microstructural changes and the formation of agglomerates.^47,48^

Compatibility between the reinforcement and the polymer matrix is a critical factor for the material’s performance. Although both lignin and CNC are derived from biological sources, the structural heterogeneity of lignin and the high crystallinity of CNC can present challenges to dispersion and the formation of a homogeneous network at high concentrations. Functionalization of the lignin surface is a known strategy to increase the interaction with Poly isocyanates, which can improve the dispersion and cross-linking density of the foam.^49^ The way the CNC interacts with this functionalized lignin therefore becomes a determining factor for the *T*_g_ behavior and overall performance of the material.

Figure 5 shows mass gain due to water absorption, compared to pure RPUF, loadings of 0.2%, 0.4%, 0.8% CNC produce a decrease in water uptake behavior. According to Gu et al.,^50^ when chemically compatible fillings are incorporated into the RPUF system, new small bubbles are formed, which can encapsulate the blow of gas and compromise the filling of the spaces with water, thus having less space for water to enter. Furthermore, Członka et al.^51^ stated that the morphological characteristics of the cellular structure of RPUF are the main influencing factor due to their hydrophobic/hydrophilic character.

**Figure 5:**
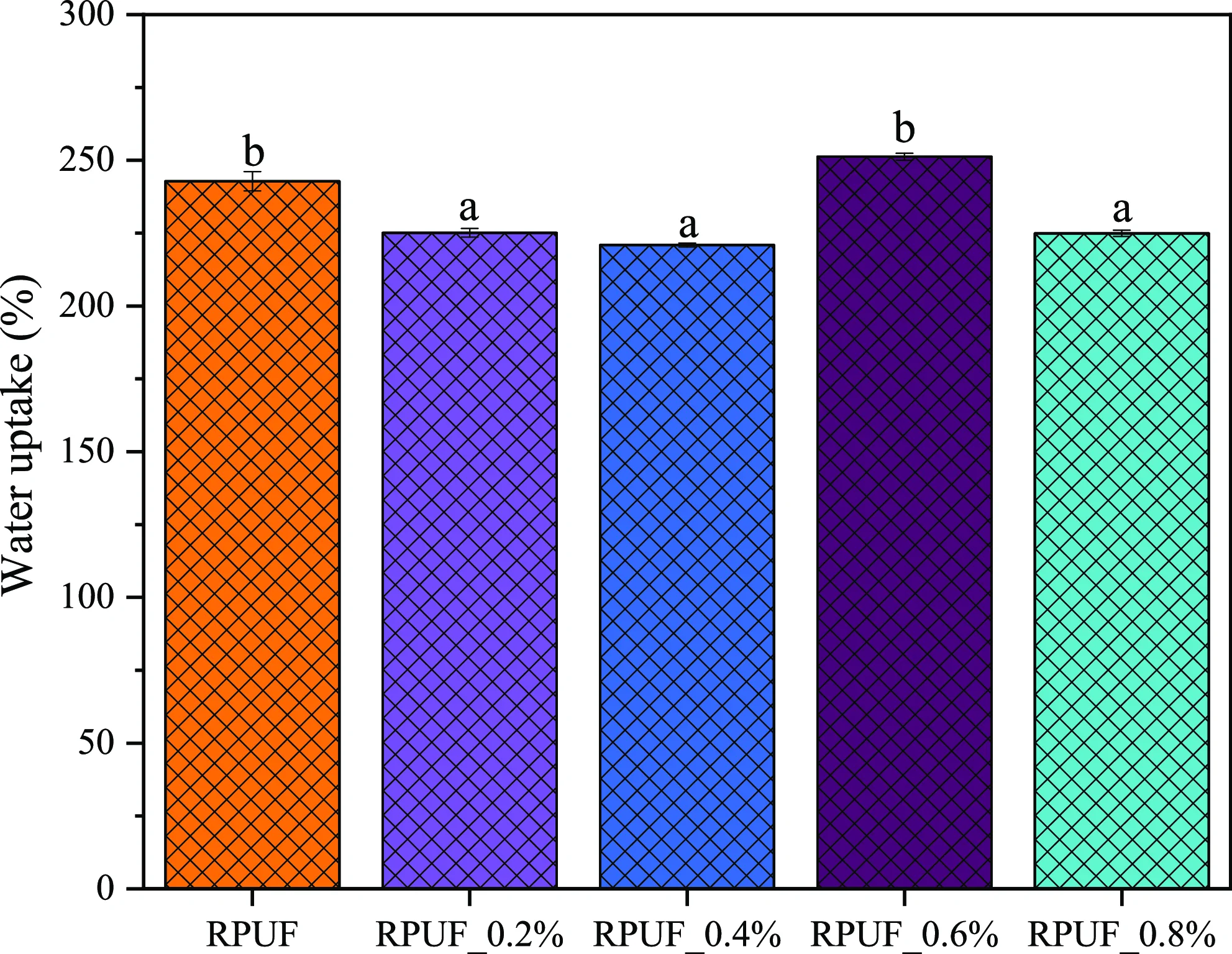
Water uptake of the lignin-based RPUF (Different letters above the bars represent statistically different means).

In this sense, the anisotropic cells transmitted by the fillings can also be influenced by the water uptake results obtained.^8^ All in all, even a similar moisture uptake can be considered a favorable result for a filled RPUF, as absorbed water can have a detrimental effect on the RPUF’s mechanical, thermal and acoustic properties.

The SEM images shown in Figure 6 indicate that the incorporation of 0.2% and 0.4% CNC produced a more regular cell structure compared to the 0.6% and 0.8% contents. The reduction in cell size observed at the lowest levels is compatible with the action of the particles as sites of heterogeneous nucleation. In polyurethane foam systems, the presence of a dispersed solid surface reduces the free energy required for the formation of a critical gaseous nucleus compared to homogeneous nucleation, increasing the amount of nuclei formed and limiting individual cell growth.^18,20^ Pardo-Alonso et al.^18^ directly demonstrated the nucleating effect of nanoparticles during the expansion of polyurethane foams, while Merillas et al.^20^ showed that the efficiency of this process also depends on the trapped air and the interfacial conditions associated with the particles.^18,20^

**Figure 6:**
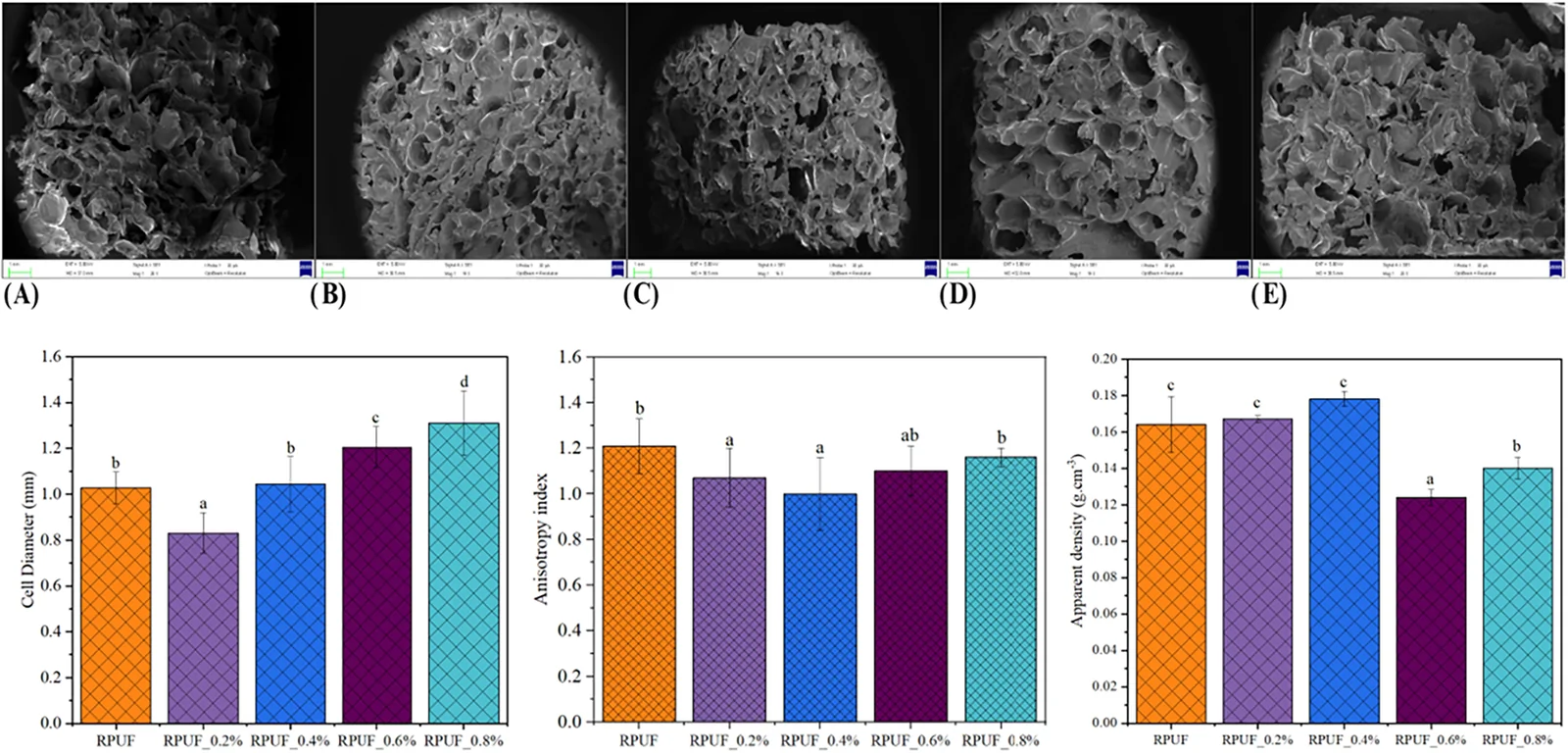
SEM images and morphological properties of the lignin-based RPUF: (A) RPUF, (B) RPUF_0.2%, (C) RPUF_0.4%, (D) RPUF_0.6%, and (E) RPUF_0.8% (different letters above the bars represent statistically different means).

The ability of the CNC to participate in this process depends on its interaction with the polymer phase and its distribution in the precursor mixture. Chen et al.^52^ demonstrated that CNC’s hydroxyl groups establish hydrogen bonds with polyurethane polar groups, forming physical cross-linking points and increasing interfacial interaction between phases. Yang et al.^53^ showed that dynamic interactions between nanocellulose and polyurethane promote structural reconstruction and the formation of physical networks in the material. Similarly, Wang et al.^31^ attributed the coupling between CNC and aqueous polyurethane to the formation of multiple hydrogen bonds at the interface, while Xu et al.^32^ demonstrated that hydrogen bond networks contribute to the reorganization and stabilization of polyurethane structures.

The changes in the O–H, N–H and C=O bands shown before in the FTIR support the occurrence of interactions between the hydroxyl groups of the CNC and the polar groups of the polyurethane matrix. These interactions favor the integration of particles into the polymeric phase and help explaining why the lower CNC contents produced a more uniform cell structure. The influence of intermolecular interactions on the organization of porous structures was also demonstrated by Zeng et al.,^33^ who attributed the reorganization of cellulose hydrogen bonds to molecular assembly and the formation of the porous structure. At 0.6%–0.8% content, the higher cell heterogeneity indicates reduced nucleation efficiency.

Table 2 quantifies the cellular refinement, which was most pronounced for the lowest CNC content (RPUF_0.2%), for which the mean cell dimension was 0.831 mm, compared to 1.027 mm for neat RPUF, corresponding to a reduction of ca. 19.1%. The projected cell area, that incorporates both cell length and height, decreased from 0.844 to 0.601 mm^2^, representing a reduction of ca. 28.8%.

**Table 2:** Cell Dimension and Projected Cell Area of the Lignin-Based RPUF^a^

	Cell dimension (mm)	Projected cell area (mm^2^)
RPUF	1.027 ^(0.061)b^	0.844 ^(0.097)a^
RPUF_0.2%	0.831 ^(0.086)a^	0.601^(0.127)b^
RPUF_0.4%	1.045 ^(0.124)b^	0.981^(0.231)c^
RPUF_0.6%	1.205 ^(0.083)c^	1.183 ^(0.167)b^
RPUF_0.8%	1.314 ^(0.142)d^	1.519 ^(0.357)d^

a Different letters within the same column indicate statistically significant differences according to Tukey’s test (*p* < 0.05).

Nevertheless, further increases in CNC content resulted in progressively larger cells, with projected areas of 1.183 and 1.519 mm^2^ for RPUF_0.6% and RPUF_0.8%, respectively. The larger standard errors observed for the higher CNC contents, particularly for RPUF_0.8%, also indicate greater variability in cell dimensions, being consistent with the more heterogeneous morphology observed in the SEM micrographs.

In addition, changes in cell size and cell shape cause changes in RPUF density, as shown in Figure 6 C. Density plays an essential role in many applications, since RPUF are widely known for their high specific mechanical properties. In addition, bulk density (Figure 6) is an indirect measurement of void content, and the higher the void content, the higher the gas content and the higher the insulation properties [32].

Considering that some recent studies have attributed increases in RPUF density to similar fillings being incorporated into RPUF [32], in this study, the fillers inserted probably altered the density of the RPUF due to its low content (CNC 0.2% wt.). Figure 7 compiles the mechanical compressive properties of the studied RPUF and Table 3 compiles the specific compressive properties of the studied RPUF. As expected, the bulk density increased with the addition of CNC up to a certain percentage, correlating with the observed reduction in cell size and the increase in cell uniformity, and this can also be seen in all the mechanical properties.

**Figure 7:**
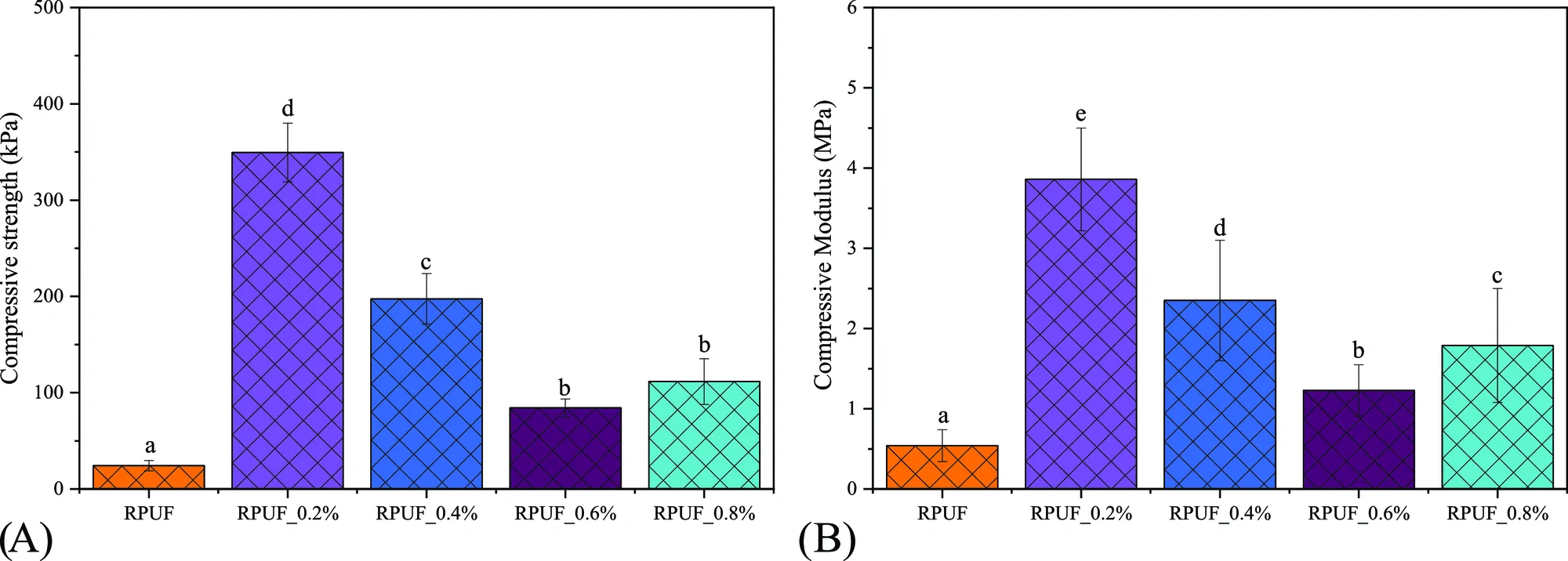
Compressive properties of the lignin-based RPUF: (A) compressive strength and (B) compressive modulus (different letters above the bars represent statistically different means).

**Table 3:** Specific Compressive Properties of the Lignin-Based RPUF^a^

	Specific compressive strength (kPa.cm^3^.g^–1^)	Specific compressive modulus (MPa.cm^3^.g^–1^)
RPUF	147.0 ^(5.3) a^	3.37 ^(0.20) a^
RPUF_0.2%	2091.9 ^(30.5) e^	23.11 ^(0.64) d^
RPUF_0.4%	1105.3 ^(26.3) d^	13.20 ^(0.75) c^
RPUF_0.6%	677.4 ^(9.4) b^	9.91 ^(0.32) b^
RPUF_0.8%	797.1 ^(23.7) c^	12.78 ^(0.71) c^

a Different letters within the same column indicate statistically significant differences according to Tukey’s test (*p* < 0.05).

These results are confirm studies such as Park et al.,(2016); Zhang et al., (2019),^9,54^ which highlight how reinforcement helps the physical and mechanical properties of PU foam. Septevani et al., (2017),^26^ in a study of the use of cellulose nanocrystals at 0.2%, 0.4% and 0.8% as a nucleating agent in foam, analyzed the mechanical properties of compression and modulus and found the same reduction in properties at 0.8% and even lower results than RPUF_0.2%.

CNC may restrict foam overexpansion and modify the cellular architecture, contributing to changes in apparent density and mechanical performance. The density-normalized compressive properties presented in Table 3 indicate that the improvement was not exclusively caused by changes in apparent density. RPUF_0.2% exhibited a specific compressive strength of 2091.9 kPa·cm^3^·g^–1^, compared with 147.0 kPa·cm^3^·g^–1^ for the neat RPUF, while the specific compressive modulus increased from 3.37 to 23.11 MPa·cm^3^·g^–1^. The formulations containing 0.2 and 0.4 wt % CNC showed the highest specific mechanical performance, suggesting a more efficient cellular structure, with improved stress distribution. At higher CNC contents, the decrease in specific strength and modulus may be associated with greater cellular heterogeneity and CNC aggregation. Nevertheless, the 0.6 and 0.8 wt % formulations still showed higher specific properties than neat RPUF, indicating improved mechanical efficiency.^55–57^

These interactions promote better dispersion of the CNC, mechanical reinforcement and increased thermal stability, as well as influencing the organization of the phases and the crystallization of the soft segments of the polyurethane.^41,58^ The presence of CNC can act as a nucleating agent, modify the microstructure and increase compressive strength and elongation, as well as improving properties such as hydrophobicity and resistance to degradation.^41,44,58^

Figure 8 and Table 4 show the log of E’ and show a glassy region up to 50 °C and then a glass transition region (50–75 °C). The first range is due to the insertion of CNC, increasing the storage modulus, producing a bond between the interface and the matrix, increasing its rigidity. This characteristic is noticeable in samples with a higher percentage of nanocellulose.^59^ The presence of CNC probably caused an increase in the stiffness of the material due to the good bond between the reinforcement and the matrix. In the glass transition region, the storage modulus graph corroborates with the Tan δ graph, showing the material’s *T*_g_ range.

**Figure 8:**
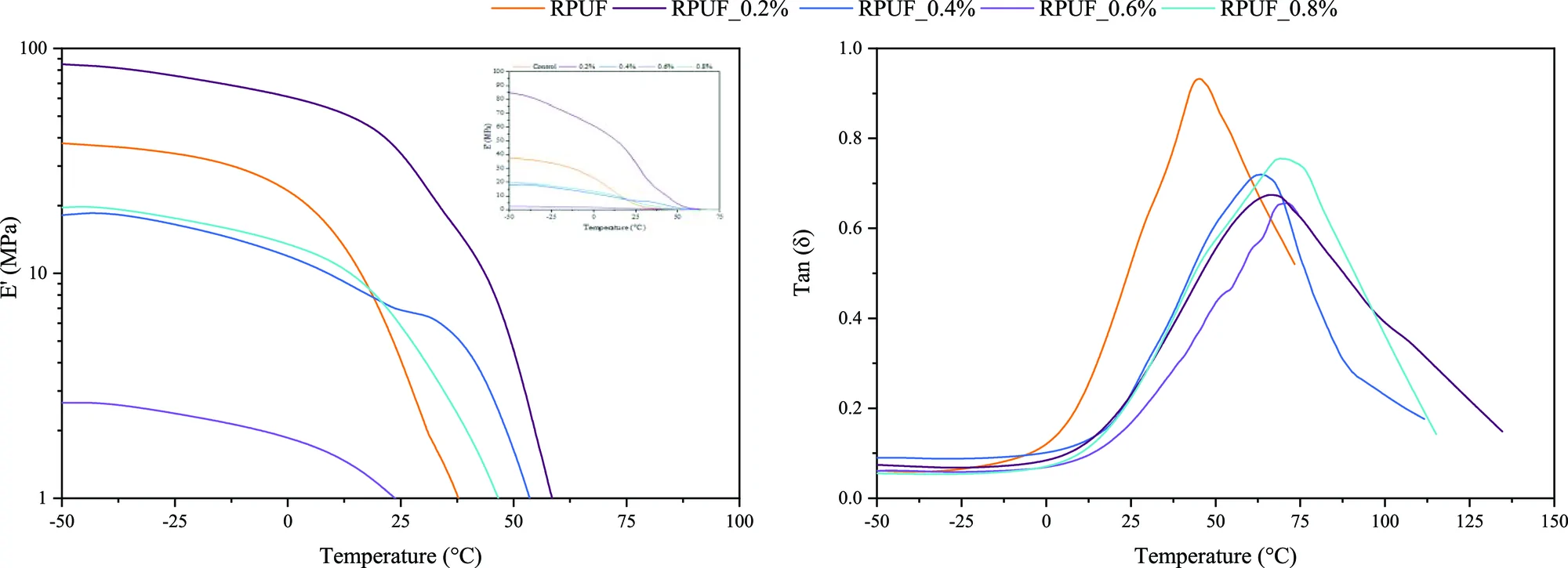
DMA curves of the lignin-based RPUF.

**Table 4:** DMA Properties of the Lignin-Based RPUF

	E’g (−50/45 °C) (MPa)	E’r (50/75 °C) (MPa)	*T*_gDMA_ (°C)^a^	Tan delta peak height	*C*
RPUF	37.82	1.02	45.4	0.93	—
RPUF_0.2%	49.84	20.21	66.8	0.67	0.07
RPUF_0.4%	18.12	6.23	62.8	0.71	0.08
RPUF_0.6%	2.65	0.68	71.0	0.65	0.11
RPUF_0.8%	19.59	3.42	71.7	0.75	0.15

In the Tan δ graph, the main information obtained is the *T*_g_, represented by the peak in the graph,^60^ in which it is possible to notice an increase of up to 10 °C when compared to the control foam, corroborating the previous DMA analysis and other studies such as.^61^

The *C* parameter is associated with the decrease in modulus when the temperature rises and the material passes through its glass transition region. The *C* value of the CNC-reinforced RPUF increased as the percentage of reinforcement increased. According to Ornaghi et al. (2010),^62^ the lower the *C*, the more effective the reinforcement, which can be attributed to improved reinforcement/matrix interaction related to overpacking.

Regarding the height of the tan delta peak, again, overpacking produced a decrease of ∼22% overall in all RPUF significantly, which means lower damping properties. Furthermore, according to Hejna et al. (2017),^63^ a sharp tan delta peak indicates a sharp distribution of relaxation times.

According to Chang et al. (2001),^64^ the tan delta curve is the best criterion for calculating *T*_g_, since it represents the midpoint of the viscoelastic transition of the RPUF, and this methodology was chosen in this study. Despite the low CNC content, the filler promoted a slight increase in *T*_g_ values, especially for the lower percentage RPUF such as 0.2% and 0.4% which increased around ∼20 °C and ∼30 °C for the 0.6% and 0.8% reinforcements, which can be attributed to a favorable interfacial interaction between the filler and the PU which decreased the mobility of the polymer chains.^65^ Previous studies have reported similar increases in *T*_g_ for cellulose nanocrystals.^65^

## 4 Conclusions

CNC was successfully used to reinforce and improve the properties of lignin-based rigid polyurethane foams. FTIR confirmed hydrogen bonding between CNC hydroxyl groups and urethane linkages, supporting good interfacial interaction. SEM analysis revealed that the incorporation of CNC promoted a finer and more homogeneous cell morphology, with an average cell size reduction of up to 18% at 0.2–0.4 wt.% compared to neat RPUF. The CNC-containing foams exhibited higher specific compressive properties than the neat foam, particularly RPUF_0.2%. These results indicate that the mechanical improvement was not related only to differences in apparent density but also to CNC-induced changes in the cellular structure of the foam.

Thermogravimetric analysis showed a shift in the temperature at 10% mass loss from 272.6 °C (control) to 282–285 °C at 0.2–0.4 wt.% CNC, while DSC revealed an increase in *T*_g_ from 31.5 °C for the neat RPUF to a maximum of 58.2 °C for RPUF_0.2%. Water uptake was also reduced for CNC-containing foams, indicating improved dimensional stability. At higher CNC concentrations (≥0.6 wt.%), aggregation effects led to irregular cells and a slight decrease in performance, confirming that the optimal nucleating effect occurs at lower loadings.

Overall, these findings demonstrate that the observed reduction in cell size and increase in estimated cell density are consistent with a heterogeneous nucleation effect promoted by CNC in lignin-based RPUF, promoting cell refinement, higher cell density, and enhanced thermo-mechanical performance, while maintaining sustainability advantages. Nevertheless, more direct evidence of nucleation would allow a more rigorous assessment of the role of CNCs in the nucleation mechanism.
